# Are Dementia Patient's Engagement Using Tailored Stimuli the Same? The Apathy Dilemma in Nursing Home Residents

**DOI:** 10.1155/2012/942640

**Published:** 2012-08-26

**Authors:** Elsa Leone, Audrey Deudon, Julie Piano, Philippe Robert, Arnaud Dechamps

**Affiliations:** ^1^Le Centre Mémoire de Ressources et de Recherche, Centre Hospitalier Universitaire de Nice 06000 Nice, France; ^2^Laboratoire d'Anthropologie et de Psychologie Cognitives et Sociales, Université Nice Sophia Antipolis 06100 Nice, France; ^3^EA CoBTek, Université Nice Sophia Antipolis 06100 Nice, France; ^4^Plateforme Patients du Centre Hospitalier et Universitaire de Nice, Nice 06100, France; ^5^Department of Psychology, University of Pancasila, Jakarta 123930, Indonesia; ^6^CobTeK/CMRR, Pavillon Mossa, Rez de Jardin Hôpital de Cimiez 06000 Nice, Nice 06000, France

## Abstract

*Background*. Apathy is the most frequent behavioural disturbance understanding how apathy drives engagement in resident's activities of interests is a milestone to better understanding and tailored challenging interventions targeting engagement enhancement. *Method*. Residents aged 60 and older with dementia according to the ICD 10 from four nursing homes in the south east of France. A set of 25 stimuli were used and categorized by participant into Work, Leisure, Family, or Personal categories, an additional “not interested” category was used for comparison of engagement. The participants stimuli allocation was randomized in guided and unguided situations over a two-week period with 15minute interaction for each stimulus (*n* = 2) of each category (5×(15 min×2)). Clinical trial identifier: NCT01314131. *Results*. The mean age, 95% confidence interval (CI) of the 40 participants was 85.4 (83.8–87) with a mean MMSE score, CI95% of 17.7 (16.5–19). Analyses revealed a significant superiority effect of guidance over unguided interaction in duration of engagement in all categories of interest except for the stimulus category “family” and all *P* < .05. Apathetic participants when guided had longer engagement duration in stimulus Leisure and Personal (all *P* < .01). *Conclusion*. Guidance and better activities of interest can lead to enhanced engagement time in participants with dementia.

## 1. Introduction

Nursing home residents can lead lives that may lack purposeful activity. This has been postulated as the reason for the increased levels of agitation and aggression that can occur in such settings [1–5]. Over the past few years, the concept of “engagement” has emerged as a means of understanding the behavioural pattern often seen in nursing home residents with dementia [3, 6, 7]. “Engagement” is best understood as “being involved or occupied with external stimuli” [8].

Vygotsky developed the idea of “zone of proximal development” (ZPD) in the 1930s, and this idea combined with individualized approaches to patient care has found wide acceptance in the fields of social, educational, and clinical psychology [2, 9, 10]. Clinical interventions relying on the concept of ZPD have been found to enhance participation and minimize frustration in nursing home residents [2, 9]. Engaging nursing home residents, particularly those living with dementia, in meaningful activities may have positive health outcomes [11, 12]. However, apathy and other behavioural symptoms can affect the person's ability to engage [5, 9, 13]. Apathy, a disorder of motivation [14], is the most frequent neuropsychiatric symptom observed in dementia, regardless of disease stage [15]. Apathy is usually described as being a lack of interest in the initiation of and response to social interaction [16]. Ironically, apathy is considered one of the least distressing neuropsychiatric symptoms of those assessed by the Neuropsychiatric Inventory [4]. Despite its ubiquity, the impact of apathy in the lives of nursing home residents is far from clear, and although interest and engagement decrease with age [17], the extent to which this is a function of apathy is still unknown. The purpose of this study is to explore the role of apathy in people living with dementia and to determine whether or not specific or “guided” interventions may improve engagement. Based on the literature [2, 3, 18], we hypothesized that the level of interest in a particular activity would determine the level of engagement of residents, regardless of the level of apathy and whether or not the intervention was “guided.” 

## 2. Method

This study was a multicentre single-arm trial comparing the efficacy of “guided” versus “nonguided” interactions on the level of engagement during individualized activities in nursing home residents. This study was approved by the Sud Mediterannée IV ethics committee and all participants gave their consent for this study. 

## 3. Participants

**Figure 1:**
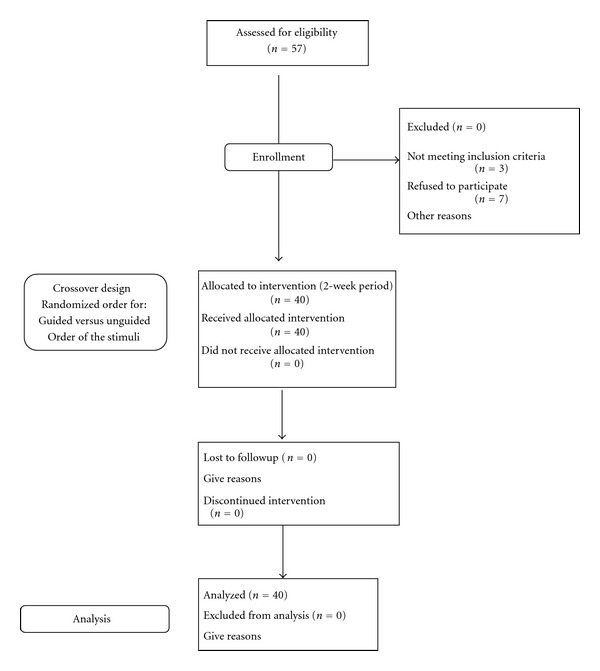
Participants flowchart.

Fifty-seven residents from 4 nursing homes in the South East of France were screened for eligibility to participate in the study. Of these, 40 met inclusion and exclusion criteria enrolled in the study.  Figure 1 shows the flow of the patients. The nursing homes which agreed to participate are part of a research the French research network, Réseau EHPAD Recherche (RER). Inclusion criteria were (1) a diagnosis of dementia according to ICD 10 criteria [19]; (2) age 60 and older; (3) living in a nursing home; (4) minimental State Exam (MMSE) score between 10 and 26; (4) being able to respond to basic commands; and being able to sit in a chair or wheelchair. Noninclusion criteria were: (1) residents below 60 years of age, (2) unable to answer to basic orders, (3) having aphasia, (4) motor or functional limitation impeding all interactions and occupational activities, or (5) unable to sit on a chair or wheelchair. Participants were regrouped in the apathetic group when they expressed a score equal or superior to 3 at the apathy inventory (AI).

### 3.1. Randomization

The order of stimuli presented and whether or not the sessions were “guided” was randomly assigned through a computerized allocation matrix. Therapists and assessors were blind to the study objectives as well as the randomized allocation scheme for stimuli presentation order. 

## 4. Procedure

The intervention was comprised of two parts: (1) establishing a list of activities in which each participant expressed an interest; (2) presenting stimuli for each type of activity in which the participant expressed an interest. For the first part, the list of activities of interest was established by administering an electronic version of a previously developed intervention called the “test of interests” (TILT) to each participant. The TILT was modified to enable administrations using an electronic tablet (iPad) and is available online at www.cmrr-nice.fr. In the TILT, participants were shown 40 images of selected activities and asked to whether each activity interested them or not (Table 1). To enhance understanding, the interviewer prompted the participant by asking, “are you interested in this?” and then named the activity. If the answer was “no”, then the next activity was presented. If the answer was “yes”, the interviewer added follow-up questions about the activity according to a set script. This was done in order to classify the activity into one of four preselected categories (Work-occupation/Personal/Leisure/Family) or “I do not know” according to the Cohen Mansfield procedure [20]. Once the script was completed, the next activity was presented. The therapist ensured correct understanding of the question and aided the rrsidents during the process. A standardized two-hour training procedure for the interviewers was developed to ensure proper use of the material and reproducibility of the assessments. 

### 4.1. Selection Procedure for the Individualized Activities

After the list of activities was collected, the 40 images were clustered into groups. This clustering was done using the feedback of a focus group convened for this purpose and composed of participants, carers, family members, and a neurophysiologist (*n* = 30) (Table 1). This resulted in a final list of activities which was then presented to another focus group composed of different participants and their carers (*n* = 32). This second group was then asked to choose two objects from a list of six objects corresponding to each activity (*n* = 25). The objects were stimuli that would be implemented during the intervention (Table 1).

The results were then matched to the reduced list of activities (*n* = 25) in order to choose the corresponding stimuli for intervention. The selection of stimuli for the four groups of interests and “not interested” was based on the highest and lowest reported interests, respectively. For each participant 4 × 2 stimuli of interest and 2 of “not interested” were presented in random order over a period of two weeks, giving a total of 10 sessions of approximately 45 minutes. Each category presentation lasted for a maximum of 15 minutes with the 2 stimuli manipulated during each session. 

The “guidance” involved was the presence of a psychologist who informed the participant about the stimulus and invited them to use it and talk about it. The therapist first indicated the name of the chosen activity and reminded the participant that they had described this activity as “interesting” during the preceding visit. The therapist also reminded the participant of which category they had previously assigned to the activity. In order to enhance interaction with the participant, the therapist helped them recall the information and memories that they had mentioned during the first interview. Finally, the therapist showed the activity picture on the iPad and named the two stimuli used for the activity, before manipulating them in front of the subject. Then the participant was asked to interact with the object and the resulting engagement time was recorded. When participants were not being “guided” the therapist remained in the room but did not interact or interfere with the participant.

## 5. Assessments

Demographic information including age, sex, level of education, and clinical diagnoses were obtained from the medical records for each participant. 

### 5.1. Primary Outcome Measure: Observational Measurement of Engagement (OME)

The primary outcome measure consisted in changes in the “observational measurement of engagement (OME)” during the intervention session. We followed the OME procedure described in previous studies [8, 20]. OME data were recorded using a paper-based version. The assessor recorded both duration of engagement and level of attention during the session. The specific outcome variables on the OME are as follows.

“Duration” referred to the amount of the time that the participant was engaged with the stimulus. This measure started after presentation of the stimulus and ended at 15 minutes, or whenever the study participant was no longer engaged with the stimulus (i.e., the study participant was no longer observed to exhibit attention behaviours for 30 s.) Duration was measured in seconds.

“Attention” to the stimulus during a session was measured on a 4-point scale: not attentive, somewhat attentive, attentive, and very attentive. Attention could be gauged based on the following: the amount of attention the person was visibly paying to a stimulus during the session (e.g., eye movements, manipulating or holding the stimulus, talking about the stimulus), and whether the person was following instructions provided (e.g., how to build with the blocks). Attention could also include physical manifestations without visual contact (e.g., touching an object, even if looking away) [8]. 

“Attitude” to the stimulus during a session was measured on a 7-point scale: very negative, negative, somewhat negative, neutral, somewhat positive, positive, and very positive. Attitude was typically determined by gauging the amount of excitement and/or expressiveness toward the stimulus (e.g., smiling, frowning, energy, excitement in voice). We recorded attitude to the stimulus seen during most of the trial as well as the highest rating of attitude observed during the trial [8]. The authors reported an interrater reliability of the OME of 0.78 for the engagement outcome variables [8]. 

### 5.2. Secondary Outcome Measures

Behavioural disturbance was evaluated using the neuropsychiatric inventory nursing home version (NPI-NH) [21]. The NPI-NH consists of a 15–20-minute interview by a psychologist involving at least two staff members who usually care for the resident (range from 2 to 7 staff members including a certified nurse). The NPI-NH has 12 neuropsychiatric domains which are delusions, hallucinations, agitation/aggression, depression/dysphoria, anxiety, euphoria/elation, apathy/indifference, disinhibition, irritability/lability, aberrant motor behaviour, nighttime behavior, and eating disorders. The NPI score ranges from 0 to 144 and higher scores indicate greater behavioural disturbances. 

“Apathy” was evaluated using the apathy inventory (AI) caregiver version [22]. The AI assesses three dimensions of apathy including emotional blunting, which refers to the lack of emotional responses; lack of initiative, which refers to diminished goal-directed behaviour; lack of interest to diminished goal-directed cognition. The Caregiver version follows the rules and structure of the NPI and scores range from 0 to 12 with a cutoff score of 3 indicating the presence of apathy. 

The MMSE was used to assess cognitive status and for statistical adjustment purposes only. The severity of dementia was categorised into stages according to ranges of MMSE scores as follows 21–26: mild, 16–20: moderate, and 10–15: moderately severe [23].

The frontal assessment battery (FAB) is an instrument that helps to distinguish Alzheimer-disease (AD) from other frontal-type dementias characterized by dysexecutive function. The maximum total score is 18 with higher scores indicating better performance [24]. FAB data are presented for descriptive purposes only.

## 6. Analysis

Distribution of plots was performed on each variable, mean, 95% confidence intervals were presented, when normal distribution was not observed, nonparametric tests were performed (Wilcoxon test). Differences between groups were normalized using *Z* score (one standard deviation from the median). Multivariated models and linear regressions were used using by a stratified method for confounding variables. 

All analyses were conducted on an intention-to-treat basis, using all available data from all patients and carrying forward the last observation for dropouts or missing data. No missing data was found at baseline or during interventions. All *P* values were adjusted using Bonferroni correction. 

## 7. Power and Sample Size

Based on the hypothesis that guidance would produce better engagement time, we determined using G*Power and that a sample of 40 subjects using crossover design would produce 90% power to detect a difference of 80 ± 91 seconds with 25% chance of lost to follow-up and alpha set at 5%.

## 8. Results

### 8.1. Baseline Characteristics

The mean age with 95% confidence interval (CI) of the 40 participants was 85.4 [83.8–87] with a mean MMSE score, CI 95% of 17.7 [16.5–19] (Table 2). Participants were predominantly female (72.5%). All participants had dementia based on their medical records and further testing by the research team when necessary. The breakdown of diagnoses was as follows: 18 participants had Alzheimer, disease, 8 Mixed Dementia, and 14 had other dementia types (including vascular dementia, fronto-temporal dementia).

The interest frequency for each activity (*n* = 40) is presented in Table 1. Data from the observational survey (work submitted) is presented for comparison. Of the 40 activities (Table 1) presented to each participant, “Enjoying a good meal,” “Dressing up,” “Reading,” “Watching TV,” “Museum,” “Tourism,” and “Entertainment” were found the most interesting by 80% or more of the population. These results slightly differ from the survey (*n* = 601, work submitted), yet, most of the observed interests of the participants (*n* = 40) are equivalent to the survey. 

Only Apathy was found to influence the OME scores. There was no significant demographic and clinical characteristics difference between Apathetic and non-Apathetic patients at baseline (Table 2). 

### 8.2. Guided versus Nonguided

Analyses of the overall population revealed that duration of engagement was significantly increased during “guided” sessions for all categories examined except for “Family” *P* < .05 (Table 3). We did not find any difference in the category “not interested” between guided and unguided sessions. “Attitude”, followed by “Attention” showed a positive trend during the guided sessions especially for the Work, Family, and Leisure stimuli with Z score ranging from −2 to −2.8 compared to unguided sessions (Table 3).

### 8.3. The Interaction of Apathy and Engagement

The presence of apathy was found to reduce length of engagement in unguided sessions, in stimuli related to Leisure, Personal, and “Not Interested” with Z score of −2, *P* = .004, −3, *P* ≤ .001, and −2.5, *P* < .001, respectively. Nonapathetic participants had longer engagement time when guided except for the stimuli “Family,” and “Not Interested” (Table 4). Apathetic participants had their longest engagement duration when guided with stimuli “Leisure” and “Personal” (all *P* < .01). In non-apathetic participant guidance and nonguidance were equivalent in attention and attitude in all circumstances except for stimuli work and family (*P* < .02), where guidance revealed higher scores.

### 8.4. Impact of Stimulus in Interested versus Not Interested

We found duration of engagement and attitude scores were higher in all circumstances in all four stimuli categories when residents were experiencing stimuli of interest to them compared to when stimuli, not of interest (Table 5). 

Attention results showed that apathetic participants showed improved “Attention” in both guided and unguided sessions in all stimuli of interest compared to when stimuli were not of interest (all *P* < .05) (Table 5).

## 9. Discussion

This study suggests that for both apathetic and nonapathetic people living with dementia, guided individualized interventions based on participant's interests lead to improved levels of engagement and attitude. Our results show that guided interaction increased engagement duration for some activities (in personal, leisure, and work categories). Our study confirms numerous data on the relatively important role to health of guidance and of tailoring the content of interaction to the participant's characteristics and needs [2, 6, 9, 10, 23, 25] and we have highlighted the negative impact of apathy in participation and level of engagement in nontailored interaction.

Among the diagnostic criteria of apathy [26], criterion B describes the three core clinical domains: reduced goal-directed behaviour; goal-directed cognitive activity; and emotions. Each domain includes two symptoms: the first symptom pertains to self-initiated or “internal” actions, cognitions and emotions; and the second symptom to the patient's responsiveness to “external” stimuli. A validation study of the criteria [5] indicated a higher frequency of the initiation symptoms, whereas the responsiveness capacities were less affected. Also, the lack of social and physical activity observed in residents in nursing homes are often viewed negatively in terms of health maintenance and quality of life [2, 11]. Our results depicted a reduction of active social participation in nursing home residents. Furthermore, we found that for apathetic participant the previous meaning associated with some social activities was lost. 

Interestingly we found that the “Family” stimulus showed equivalent results for both apathetic and nonapathetic participants either when guided or not. Apathetic participants responded best to being guided when exposed to stimuli of the Personal or Leisure categories rather than for the Work or Family categories. Nonapathetic participants also responded best to stimuli in the Personal and Leisure categories and those responses were better than the corresponding responses for apathetic participants. Nonapathetic participants also benefited from guided interaction to stimuli in the Work category. 

There may be many reasons for these results. However, the observed equivalence of outcome in both the social (Work/Family) categories could underpin a plausible nosological social identity deconstruction pattern for apathetic participants. Social identity, as a systemic and personality construct, is mediated by the interaction of the self (individual) with the outside world (other or groups) [27]. The feeling of being part of, and involved in a group is conditioned by the various experiences acquired through group affiliation [28, 29]. Accordingly, social identity created within the family structure is shaped within inner group boundaries such as experiences and feelings, which in return produce social actions within the group [28].The observed social and functional deconditioning of old adults in nursing homes [27] could contribute to the breakup of family bounds. 

As the severity of dementia increases, interventions based on Family and Work may become less meaningful for participants.Lack of meaning has been found to create ambiguity, and may be a reason for the poor results from interventions targeting behavioural disturbances based on Family and Work categories activities [9]. Even though all participants expressed interest in, and were motivated by, the idea of “Family,” they did not demonstrate the social activity related to it, which could explain why the duration of engagement was equivalent in both guided and nonguided sessions. 

Another potential explanation for this equivalence might be the nature of the stimulus used. As emotions and meanings can be understood as contextual, personal investment in an activity is determined by the meaningfulness and emotional weight that people attribute both consciously and unconsciously to the stimulus [9, 27, 30]. Although the stimuli we used were validated by patients and carers, there are bound to be differing individual meanings attributed by individual participants. 

Another area where this was manifested was in the results from the “Work” category. Apathetic participants showed equivalent durations of engagement in both guided and nonguided sessions. Apathy reduces both initiation and responsiveness [16], and it is unsurprising that the “Work” category showed the observed results as work stimuli involve high level of personal engagement, motivation, and effort to reach a goal. Interestingly, nonapathetic participants did show increased engagement duration when guided in the Work stimulus sessions.

The idea that meaningfulness is an important determinant of engagement for apathetic participants is supported by the data on the “Attitude” score in the guided sessions. This reflects the nosological construct of apathy in terms of changes in emotional, behavioural, and cognitive responsiveness [5]. Nonapathetic participants had a much more positive response on the “Attitude” domain, particularly in the “Work” and “Family” guided sessions. Thus, in nursing homes residents who live with dementia, it is the apathy component of the condition that determines levels of engagement. There is very little in the published literature that can aid staff in nursing homes to help apathetic residents, or to allocate resources optimally. Unfortunately, there is a limit to the conclusions that can be drawn from a small study such as ours. However, the large *Z* scores we found are impressive. This is despite the fact that we were unable to obtain all the demographic information on participants that we would have liked to obtain, and in particular in relation to time of onset of the condition. We did find some distinct neuropathological differences between apathetic and nonapathetic participants. 

In summary, both apathetic and nonapathetic participants showed benefits from being guided in their interactions with the various stimuli, although unsurprisingly nonapathetic subjects responded more positively. Furthermore, tailoring the activities to the individual circumstances of the subjects further increased the effectiveness of the stimuli involved. It is also worth noting that the corollary is true in that nonstimulation increases noninvolvement, particularly in apathetic. A key role of staff in nursing homes is to overcome apathetic subjects' unwillingness to participate in activities to help break that downward spiral [1].

## Figures and Tables

**Table 1:** List of 40 images of activities included in the TILT. Interest occurrence for the survey and the intervention study. *Data from phase one from the protocol, involving 601 surveyed participants from nursing homes in France (work submitted).

Interest occurrence in percentage and number of participants					
Stimuli used as stimulus for intervention	Interest proportion by activity	Survey (*n* = 601) *£*		Intervention (*n* = 40)	
(based on focus groups validation)		%	*N*	%	*N*
Pan/stove	Enjoying a good meal	83	499	80	32
Cooking book/food retailer catalogue	Cooking	60.4	363	47.5	19
	Shopping delicatessen	41.6	250	27.5	11
Bowtie	Dress up	75.9	456	85	34
Clothes catalogues	Shopping	49.4	297	47.5	19
Television TV program	Watching TV	75.5	454	82.5	33
Newspaper Les misérables, Victor Hugo	Reading	72	433	85	34
Chapelet/kipa/praying mat Bible/Coran/Tora	Place of worship	52.9	318	72.5	29
Museum book	Museum	59.4	357	85	34
Movie menu	Entertainment	69.4	417	87.5	35
Safety jacket	Going to the beach	67.9	408	70	28
Swimming suits	Salling	36.4	219	62.5	25
Old telephone Yellow book	Calling	63.6	382	55	22
Makeup accessories	Hair salon	61.6	370	45	18
Old Make-up magazines	Make up	36.6	220	27.5	11
Hiking book	In the mountains	60.6	364	75	30
Hiking/walking shoes	Walking	72.4	435	77.5	31
Leach Dry dogfood	Petting	61.4	369	67.5	27
Old camera Traveling book	Tourism	56.4	339	90	36
Cards Cross words	Play games	53.7	323	62.5	25
Cissors	Manual activities	52.9	318	52.5	21
Color pencil/pen	Writing	41.8	251	32.5	13
Tissue Nail/sewing cotton	Sewing	42.6	256	40	16
Gardening magazines Secateurs	Gardening	43.9	264	55	22
Tennis racket Sport magazines	Do sport	43.6	262	70	28
Shovel/broom	Dish washing	34.6	208	15	6
Sponge	Housework	41.6	250	25	10
Hammer DIY books	Do DIY	40.6	244	47.5	19
Boules Jack	Play boules	40.1	241	40	16
Collection books/magazines	Hunt for antiques	29.8	179	25	10
Coins books	To collect	24.3	146	32.5	13
Harmonica Drum	Play music	29.1	175	57.5	23
Fishing hooks Hunting and fishing magazines	Fishing/hunting	22.3	134	20	8
Loto	Gambling	10.6	64	15.5	5
Game on iPad	Play video games	6.3	38	2.5	1
Laptop Mouse	Computer	10.5	63	15	6
Not used during the intervention	To rest	70.2	422	70	28
	Kissing	57.1	343	67.5	27
	Grand children gathering	73.2	440	62.5	25
	Family pictures	72	433	77.5	31

**Table 2:** Participant's demographics and clinical characteristics.

Characteristics	Apathetic, *n* = 14	Nonapathetic, *n* = 26	*P* value	Total (*n* = 40)
Age, *y*, mean, CI95%	83.6 (81.2–86)	86.3 (84.2–88.5)	NS	85.4 (83.8–87)
Gender, female, *n*, %	8 (57)	21 (81)		19 (72.5)
Education, *n*, %				
Primary school	8 (20)	17 (42.5)	NS	25 (62.5)
High school	6 (15)	2 (5)	NS	8 (20)
Tertiary qualification (TQ)	0 (0)	7 (17.5)	NS	7 (17.5)
Mini mental state examination score (0–30), mean, CI95%	17.9 [15.9–20]	17.6 [16–19.1]	NS	17.7 [16.5–19]
Moderately severe cognitive impairment (10–15), *n* (%)	5 (12.5)	12 (30)	NS	17 (42.5)
Moderate cognitive impairment (16–20), *n* (%)	6 (15)	8 (20)	NS	14 (35)
Mild cognitive impairment (21–26), *n* (%)	3 (7.5)	6 (15)	NS	9 (22.5)
Neuropsychiatric inventory (0–144), mean CI95%	19.7 [9–30.5]	9.5 [6.1–13]	NS	13.1 [8.7–17.5]
Neuropsychiatric distress Total (0–60), mean CI95%	6.5 [2–11]	4 [2.8–5.3]	NS	4.9 [3.2–6.6]
Frontal assessment battery (0–18), mean CI95%	10 [8.7–11.3]	8.7 [7.7–9.8]	NS	9.1 [8.3–9.9]
Apathy inventory criteria (0–12), mean CI95%	3.7 [2.3–5.2]	2.6 [1.6–3.5]	NS	2.9 [2.2–3.7]

Note: Nonparametric and parametric tests were used according to the data characteristics.

**Table 3:** Observational measurement of engagement (OME) scores in guided and unguided conditions for the total population.

Stimulation based on participant stimulus choice for each category (*n* = 40)		Engagement duration, s	*Z*	*P* value	Attention	*Z*	*P* value	Attitude	*Z*	*P* value
Category “Work”										
Total population	Guidance Without guidance	470 388	−2.7	<.01	2.9 2.6	−.5	.6	5.3 4.7	−2.8	<.01

Category “Family”										
Total population	Guidance Without guidance	440 360	−1.8	.06	2.7 2.6	−1.1	.2	5.2 4.8	−2	.04

Category “Leisure”										
Total population	Guidance Without guidance	554 382	−3.8	<.001	3.1 2.8	−2	.04	5.6 5.2	−2.1	.03

Category “Personal”										
Total population	Guidance Without guidance	511 375	−3.1	.002	3 2.9	−1	.3	5.3 5.2	−.9	.4

Category “Not Interested”										
Total population	Guidance Without guidance	238 228	−.6	.5	2.3 2.4	−.8	.4	4 4	−.1	.9

Note: *P* value significance is 2-tailed and is given for the *Z* score using bootstrap method. *Z* score is based on “without guidance” rank.

**Table 4:** Observational Measurement of Engagement (OME) scores in guided and unguided conditions in apathetic and non apathetic participants.

Stimulation based on participant's stimulus choice for each category in Apathetic (*n* = 14) and nonapathetic (*n* = 26) participants		Engagement duration, s	*Z*	*P* value	Attention	*Z*	*P* value	Attitude	*Z*	*P* value
Category “Work”										
Apathy	Guidance Without guidance Mean difference, 95% CI	364 332 33 [−134–182]	−.8	.4	2.7 2.4 0.4 [0.6–2.3]	−2	.046	4.6* 4.3 0.3 [−0.4–0.9]	−.7	.4
Non Apathy	Guidance Without guidance Mean difference, 95% CI	527 415 111 [22–202]	−2.6	.01	3 2.7 0.3 [0.03–0.6]	−2.1	.035	5.7 4.9 0.8 [0.2–1.4]	−2.4	.016

Category “Family”										
Apathy	Guidance Without guidance Mean difference, 95% CI	424 360 72 [−55–221]	−.5	.5	2.6 2.4 0.3 [−0.08–0.8]	−1.3	.2	4.5* 4.5 −0.08 [−0.8–0.5]	−.07	.9
Non Apathy	Guidance Without guidanceMean difference, 95% CI	448 365 83 [−24–194]	−1.8	.08	2.8 2.7 0.08 [−0.3–0.4]	−.5	.6	5.6 4.9 0.7 [0.2–1.2]	−2.4	.01

Category “Leisure”										
Apathy	Guidance Without guidance Mean difference, 95% CI	500 311* 197 [79–332]	−2.4	.01	3 2.7 0.2 [−0.08–0.5]	−1.7	.09	5.1** 4.7*0.4 [0–0.7]	−1.3	.2
Non Apathy	Guidance Without guidance Mean difference, 95% CI	583 418 165 [62–256]	−3	.002	3.1 2.9 0.4 [0–0.8]	−1.3	.2	5.8 5.5 0.4 [−0.2–0.8]	−1.8	.08

Category “Personal”										
Apathy	Guidance Without guidance Mean difference, 95% CI	426 237*200 [84–323]	−2.9	.004	2.7 2.5*0.3 [−0.08–0.8]	−1.3	.2	4.8* 4.6*0.2 [−0.4–0.8]	−.6	.5
Non Apathy	Guidance Without guidance Mean difference, 95% CI	557 444 112 [−2.9–223]	−2	.046	3.1 3.1 0.08 [−0.3–0.4]	−.4	.6	5.7 5.6 0.1 [−0.3–0.5]	−.6	.5

Category “Not Interested”										
Apathy	Guidance Without guidanceMean difference, 95% CI	240 181*66 [8–154]	−1.8	.07	2* 2**0 [−0.3–0.3]	0	1	3.7 3.70 [−0.5–0.5]	−.07	.9
Non Apathy	Guidance Without guidanceMean difference, 95% CI	237 252 −10 [−56–32]	−.3	.7	2.5 2.60 [−0.5–0.5]	−1	.3	4.2 4.20 [−0.4–0.4]	−.08	.9

Note: Apathy cut off score was based on AI score above 3 out of 12.

*Group with the lowest value compared to their counterpart without apathy, *P* < .02,

**Group with lowest value compared to their counterpart without apathy, *P* < .05.

**Table 5:** Results in engagement when compared stimuli of interest versus activities categorized as non interested by the participant.

Comparison between chosen stimuli of interest versus the stimulus categorized as “Not Interested”		Engagement duration, s, Mean difference, CI95%	*Z*	*P* value	Attention Mean difference, CI95%	*Z*	*P* value	Attitude Mean difference, CI95%	*Z*	*P* value
Stimulus category “Not Interested” in Apathetic participants										
Versus Stimuli	Guidance	240			2			3.7		
	Work	79 [−11–270]	−1.3	.2	.7[.17–1.2]	−2.2	.02	.8 [−.1–1.8]	−1.6	.1
	Family	183 [333–33]	−2.5	.01	.6[.02–1.3]	−2	.046	.8 [.2–1.4]	−2.3	.03
	Leisure	260 [117–402]	−3.1	.002	1[.5–1.6]	−2.9	.004	1.4 [1–1.8]	−3.1	.002
	Personal	185 [−.5–371]	−2.1	.03	.7[.02–1.4]	−2	.046	1 [.08–2]	−2	.046
Versus Stimuli	Without Guidance	181			2			3.7		
	Work	151 [−21–323]	−2.2	.03	.4[.09–.8]	−2.3	.025	.7 [.1–1.5]	−1.7	.1
	Family	178 [20–336]	−2.5	.01	.4[.1–.9]	−1.5	.1	.9 [.1–1.6]	−2.1	.03
	Leisure	129 [−2–261]	−2.2	.03	.7[.1–1.3]	−3.3	.001	1.3 [.9–1.8]	−2.5	.01
	Personal	55 [−4–115]	−2.2	.03	.5[.1–1]	−3.2	.001	.9 [.2–1.6]	−2.3	.02

Stimulus category “Not Interested” in Non Apathetic participants										
Versus Stimuli	Guidance	237			2.5			4.2		
	Work	173 [65–282]	−3.8	<.001	.5 [.2–.9]	−2.8	.005	1.6 [.8–2.3]	−3.3	.001
	Family	211 [128–293]	−4.1	<.001	.3 [−.04–.7]	−1.7	.09	1.4 [.9–1.4]	−3.9	<.001
	Leisure	346 [250–442]	−4.4	<.001	.7 [.3–1.1]	−3	.003	1.6 [1.2–2]	−4.2	<.001
	Personal	320 [218–421]	−4.1	<.001	.7 [.3–1]	−3.2	.001	1.5 [1–2]	−3.8	<.001
Versus Stimuli	Without Guidance	252			2.6			4.2		
	Work	157 [38–276]	−2.2	.03	.08 [.3–.4]	−.5	.6	.8 [.2–1.4]	−2.5	.01
	Family	113 [17–209]	−2.5	.01	.04 [.3–.4]	−.2	.8	.8 [.2–1.3]	−2.6	.01
	Leisure	165 [67–264]	−3	.003	.3 [.04–.6]	−4.5	<.001	1.3 [.9–1.8]	−3.8	<.001
	Personal	192 [81–303]	−3	.003	.4 [.1–.7]	−4.5	<.001	1.4 [.9–1.9]	−3.9	<.001

Note: Mean differences are calculated using boostrap method, *P* value are 2 tailed.
